# The oral-gut axis: Salivary and fecal microbiome dysbiosis in patients with inflammatory bowel disease

**DOI:** 10.3389/fcimb.2022.1010853

**Published:** 2022-10-07

**Authors:** Mohamed M. H. Abdelbary, Maximilian Hatting, Alexandra Bott, Andrea Dahlhausen, Doris Keller, Christian Trautwein, Georg Conrads

**Affiliations:** ^1^ Division of Oral Microbiology and Immunology, Department of Operative Dentistry, Periodontology and Preventive Dentistry, Rheinisch-Westfälische Technische Hochschule (RWTH) University Hospital, Aachen, Germany; ^2^ Department of Medicine III, RWTH University Hospital, Aachen, Germany; ^3^ University Medical Center for Occupational Medicine, RWTH University, Aachen, Germany

**Keywords:** oral-gut axis, salivary microbiome, fecal microbiome, inflammatory bowel disease, *Streptococcus* spp., *Prevotella* spp., *Veillonella* spp.

## Abstract

Inflammatory bowel disease (IBD) is a group of chronic inflammatory disorders that fall into two main categories: Crohn’s disease (CD) and ulcerative colitis (UC). The gastrointestinal tract extends from the mouth to the anus and harbors diverse bacterial communities. Several sequencing-based studies have identified an intestinal enrichment of oral-associated bacteria and demonstrated their ability to induce intestinal inflammation in mice, suggesting that intestinal pathobionts originate from the oral cavity, particularly members of the genus *Streptococcus*. This study aimed to investigate the composition of the salivary and fecal microbiome of IBD patients (n = 14) compared to healthy controls (n = 12) and to determine the abundance of common bacterial taxa in both niches. Metagenomic DNA was extracted from saliva and fecal samples, and the 16S rRNA gene was targeted for sequencing. Our results revealed that the overall microbial composition of saliva was significantly altered in the IBD patients compared to the control subjects (*p* = 0.038). At the genus level, *Veillonella* and *Prevotella* were highly abundant in IBD (median: 25.4% and 22.2%, respectively) compared to the control group (17.9% and 13.4%, respectively). In contrast, *Neisseria*, *Streptococcus*, *Haemophilus*, and *Fusobacterium* were associated with a healthy gut state. Regarding the fecal microbiome, the IBD group had a significantly higher abundance of *Clostridium sensu stricto 1* and *Escherichia-Shigella* (both comprising pathogenic bacteria) compared with the control group. Members of both bacterial groups have previously been shown to positively correlate with intestinal inflammation and high expression of pro-inflammatory cytokines that disrupt intestinal barrier integrity. In addition, we demonstrate that the increased abundance of *Clostridium sensu stricto 1* and *Escherichia-Shigella* has also been associated with significant upregulation of certain metabolic pathways in the feces of the IBD group, including bacterial invasion of epithelial cells. *Streptococcus* was the only common genus detected in both the salivary and fecal microbiome and represented the oral-gut axis in our study. Using culture-based methods, we isolated 57 and 91 *Streptococcus* strains from saliva as well as 40 and 31 strains from fecal samples of the controls and IBD patients, respectively. The phylogenetic tree of streptococci based on *sodA* sequences revealed several patient-specific clusters comprising salivary and fecal streptococcal isolates from the same patient and belonging to the same species, suggesting that the oral cavity is an endogenous reservoir for intestinal strains.

## Introduction

Inflammatory bowel disease (IBD) is a group of chronic, recurrent inflammatory diseases of the colon and small intestine that affect more than 6.8 million people worldwide (Jairath and Feagan, 2020). IBD disorders can be divided into two main clinical entities: Crohn’s disease (CD) and ulcerative colitis (UC). A comprehensive understanding of the pathogenesis of IBD is still elusive, but it is thought to be due to a dysregulated immune response toward environmental factors, host genetics, and microbial antigens (de Souza and Fiocchi, 2016). In addition, several studies suggest that diet influences the risk of developing IBD and can modulate disease activity through its effects on the gut microbiome (Wagenaar et al., 2021; Wark et al., 2021). For example, in a mouse model, a high-protein diet (HPD) was shown to significantly increase the abundance of *Escherichia coli*, a marker of CD, suggesting that HPD may have an impact on the development of CD by causing structural changes in the gut microbiota (Zhao et al., 2022). Dysbiosis of the gut microbiota has been steadily shown to be associated with IBD, including a reduction in diversity, depletion of bacterial taxa within the phyla Firmicutes and Bacteroidetes, and enrichment of other bacterial groups such as the class Gammaproteobacteria (Morgan et al., 2012; Zuo and Ng, 2018). Of note, the gastrointestinal tract extends from the oral cavity to the anus and harbors diverse bacterial communities that interact with each other and colonize different niches along its entire length. Although the profiles of the oral and gut microbiome are well detached due to the oral–gut barrier, several studies based on high-throughput sequencing have demonstrated intestinal enrichment of oral-associated bacteria as a potential microbial signature in IBD patients. For example, *Fusobacterium nucleatum*, a bacterium that commonly colonizes the oral cavity but rarely colonizes the intestine of healthy individuals, was shown to colonize the intestine of IBD patients, demonstrating the presence of an oral–gut microbiome axis in IBD (Brennan and Garrett, 2019; Huh and Roh, 2020). Another study revealed that CD and UC patients with postoperative relapse had a higher relative abundance of *Streptococcus* spp. in their fecal samples than patients who remained in remission (Pascal et al., 2017). In addition, several studies reported that various oral manifestations such as aphthous stomatitis, oral ulcer, dry mouth, periodontitis, and gingivitis are frequently observed in IBD patients, suggesting an association between the oral microbiota and these manifestations (Vavricka et al., 2013; Khozeimeh et al., 2021). Despite the apparent link between oral manifestations and IBD, microbiome data concerning the oral-gut axis in IBD remain limited. Few studies have examined the impact of IBD on the salivary microbiome (Said et al., 2014; Xun et al., 2018; Zhang et al., 2020; Qi et al., 2021). Although there is preliminary evidence of oral dysbiosis in IBD patients, all previous studies investigated the oral microbiome of only Asian IBD patients (three studies were related to Chinese patients and one to Japanese patients) (Said et al., 2014; Xun et al., 2018; Zhang et al., 2020; Qi et al., 2021). Therefore, further studies covering other demographic and geographic regions are required. Moreover, previous studies have only investigated the salivary microbiome of IBD patients, whereas the functional changes of the salivary microbiome and the concept of the oral-gut axis have not been explored. Hence, in the present study, we investigated the composition of the salivary and fecal microbiome and their functional changes in IBD patients compared with their age- and gender-matched healthy controls. In addition, we investigated the abundance and composition of common bacterial genera in both niches (oral-intestinal) using sequence- and culture-based approaches. We demonstrated that the salivary and fecal microbiota of IBD patients differs significantly from the microbiota of healthy controls, and we discovered a dysbiotic signature associated with specific bacterial taxa. Furthermore, we demonstrate a link between oral-gut streptococcal strains, suggesting that the oral cavity is an endogenous reservoir for intestinal strains.

## Materials and methods

### Study design, population, and sample collection

In this study, 14 IBD patients and 12 age- and gender-matched healthy controls were recruited between March 2019 and April 2021 at the RWTH Aachen University Hospital. Clinical data including the patient’s gender, age, disease type, and bowel surgery were documented and shown in **Table 1**. Exclusion criteria for patients and healthy controls were as follows: (1) being under 18 years of age, (2) treated with antibiotics eight weeks before sampling, (3) chronically infected with human immunodeficiency virus (HIV), hepatitis B virus (HBV) and/or hepatitis C virus (HCV), (4) treated with cytotoxic chemotherapy, and/or (5) suffering from cystic fibrosis. Furthermore, control subjects with gastrointestinal symptoms or diseases were excluded. This study was approved by the Ethics Committee of the RWTH Aachen University Hospital (No. EK 069/19 for IBD patients and No. EK206/09 for healthy control subjects) and conducted in accordance with the Declaration of Helsinki. All participants signed a written informed consent before sample donation.

**Table 1:** Demographic and clinical characterization of IBD patients and healthy control subjects.

	IBD Patients (n = 14)	Healthy controls (n = 12)
**Demographic characteristics**		
Male (n, %)	9, 64.3%^§^	5, 41.7%^§^
Female (n, %)	5, 35.7%^§^	7, 58.3%^§^
Age (median in years)	34^$^	37.5^$^
**IBD type (n, %)**		
Ulcerative colitis	7, 50%	–
Crohn’s disease	7, 50%	–
**Clinical data (n, %)**		
Active phase	4, 28.6%	–
Remission	10, 71.4%	–
Bowel surgery	3, 21.4%	–
**Laboratory results (median)**		
C-reactive protein (mg/L)	4.2	–
Fecal calprotectin (μg/g)	204.5	–
**Medications (n, %)**		
Anti TNF	9, 64.3%	–
Azathioprine	2, 14.3%	–
5-aminosalicylic acid	5, 35.7%	–
Steroids	1, 7.1%	–
Other (such as VDZ/UST)	4, 28.6%	–
Antibiotics	0, 0%	0, 0%

IBD, inflammatory bowel diseases; VDZ, Vedolizumab; UST, Ustekinumab, ^§^p = 0.4312 and ^$^p = 0.7518.

Saliva and fecal samples were collected from each patient and healthy volunteer in a sterile 100 ml container (Sarstedt, Nümbrecht, Germany) and a transparent stool tube (LxØ 107 x 25 mm) with a spoon and screw cap (Sarstedt, Nümbrecht, Germany), respectively. In addition, a fecal catcher (Cat #. R1101-1-10, Zymo Research, Irvine, CA, USA) was used to ensure a hygienic collection of fecal samples. The bacterial richness of saliva and fecal samples was evaluated by cultivation on tryptic soy agar with sheep blood (TSASB, Oxoid, Wesel, Germany). Due to the lower diversity and richness of the cultured bacteria, additional fecal samples were collected from P01, P15, P18, and P10, which were collected approximately three weeks, five, 13, and 14 months after the first sample, respectively. Furthermore, three fecal samples were collected from P13 (11 and 14 months after the first sample). For P01, a second saliva sample was collected three weeks after the first sample. All saliva and fecal samples were divided into aliquots and stored at -72°C for further analysis. For DNA extraction and subsequent 16S rRNA amplicon sequencing, an aliquot of each sample was sent in DNA stabilizer tubes (DNA/RNA Shield Lysis Tubes-Microbe, Cat #. R1103, Zymo Research, Irvine, CA, USA) to the Microbiome Core Facility at the ZIEL Institute for Food & Health, Technical University of Munich, Freising, Germany.

### DNA extraction and 16S rRNA gene amplicon sequencing

Metagenomic DNA was isolated from the saliva and fecal samples using a modified version of the protocol by Godon et al. (Godon et al., 1997). Briefly, 600 µl aliquots of the samples in DNA stabilization solution were supplemented with 250 µl of 4M guanidinethiocyanat and 500 µl of 5% N-laurolylsarcosine sodium salt. Followed by mixing and incubation for 1 h at 70 °C with shaking at 700 rpm. Cells were mechanically lysed by three cycles (40 s; 6.6 m/s) of bead beating with 0.1 mm glass beads in a Fast Prep-24 with a cooling adapter (CoolPrep). To remove phenols and other contaminants, the cell lysate was vortexed with 15 mg of polyvinylpyrrolidone and centrifuged at 12,000 × g and 4 °C for 3 min. A volume of 500 µl of the supernatant was transferred to a new tube, and after the addition of 5 µl of RNase (10 mg/ml), the samples were incubated at 37 °C for 20 min with shaking at 700 rpm. Subsequently, DNA purification was then performed using the NucleoSpin gDNA kit (Cat #. 740230.250, Machery-Nagel, Dueren, Germany) according to the manufacturer’s instructions. The V3–V4 regions of the 16S rRNA gene were amplified for all samples in a two-step polymerase chain reaction (PCR) using the forward primer 341F-ovh: CCTACGGGNGGCWGCAG and reverse primer 785r-ovh: GACTACHVGGGTATCTAATCC. PCR products were purified using magnetic beads (Beckman Coulter Inc., CA, USA) and pooled at an equimolar quantity of 2 nM. Multiplexed samples were sequenced on an Illumina MiSeq in paired-end (2 × 300 bp) using the v3 chemistry cartridge as previously described by Reitmeier et al. (Reitmeier et al., 2020).

### 16S rRNA gene sequence analysis and microbial profiling

Merged raw paired-end reads were processed for sequence quality assessment, chimera filtering, and clustering using the IMNGS platform (Lagkouvardos et al., 2016), which implements a UPARSE-based operational taxonomic unit (OTU) clustering algorithm that assigns high-quality sequences to the same OTUs at the 97% similarity level (Edgar, 2013). Only OTUs that occurred independently at a relative abundance of 0.25% in at least one sample of the entire saliva and fecal samples collection were considered to avoid analysis of very rare or spurious taxa, as previously recommended by Reitmeier et al. (Reitmeier et al., 2021). Phylogenetic trees of resulting representative OTU sequences were generated in FastTree (Price et al., 2009). For downstream analysis of the generated OTUs, the Rhea (Lagkouvardos et al., 2017) and microeco (Liu et al., 2021a) pipelines were used to perform normalization steps, assess the adequacy of sequencing depth by rarefaction analysis, generate taxonomic classification, and estimate alpha (within-sample) and beta (between-sample) diversity. For data visualization and plotting, Rhea and microeco pipelines used the ggplot2 package system (Wickham, 2016). The content of the bacterial metagenome was predicted from 16S rRNA gene-based microbial compositions, and functional annotations were generated from the Kyoto Encyclopedia of Genes and Genomes (KEGG) (Kanehisa et al., 2012) and MetaCyc (Caspi et al., 2016) databases using the PICRUSt2 algorithm (Douglas et al., 2020). The Basic Local Alignment Search Tool (BLAST) was used to determine the similarity between the sequences of specific OTUs and bacterial taxa from the 16S rRNA sequence database (Bacteria and Archaea), all results represent the best hits that were found on 29^th^ August 2022 (Altschul et al., 1990).

### Statistical analyses

The permutational MANOVA test (PERMANOVA) implemented in the R function Adonis from the Vegan package was used to test for differences in the salivary and fecal microbial profiles. Non-parametric ANOVA Kruskal-Wallis Rank Sum and Fisher tests were calculated for non-paired numeric input variables across selected categorical variables. Furthermore, alpha diversity was assessed using species diversity (Shannon) and species richness (Ace and Chao1) indices, while beta diversity was assessed using generalized UniFrac distances (Chen et al., 2012) and visualized by multidimensional scaling (MDS) and its non-metric version (NMDS) plots. Taxonomic differences between groups were determined by a generalized linear model based on relative abundance adjusted for confounders. The statistical significance threshold was set at *p* ≤ 0.05. P-values were corrected for multiple testing using the Benjamini-Hochberg false procedure to control for false discovery rate (Benjamini and Hochberg, 1995). All statistical analyses of demographic data (age and gender) were performed using GraphPad Prism software version 9.4.1 (GraphPad Software, LLC., San Diego, CA, USA). Four normality tests, namely the D’Agostino-Pearson test, the Anderson-Darling test, the Shapiro-Wilk test, and the Kolmogorov-Smirnov test, were performed to assess the age and gender data between the IBD and control groups. The Mann-Whitney test was used to detect differences between the ages of IBD and control groups, whereas Fisher’s exact test was used to test for gender differences among both groups.

### Isolation, identification, and *sodA* gene sequencing of oral and intestinal streptococci

To enhance/stimulate the growth of the Gram-positive bacteria, including streptococci, and inhibit the growth of Gram-negative bacteria, saliva and fecal samples were streaked on Columbia colistin-nalidixic acid (CNA) agar containing 5% (vol/vol) sheep blood (Becton Dickinson, Heidelberg, Germany) at 37°C and an atmosphere of 8% CO_2_. Sterile 10 μL inoculation loops were used to further spread the applied sample material to achieve the growth of individual colonies. In general, 24 hours proved sufficient for proper growth, while 48 hours was required in some cases. MALDI-TOF MS (Biotyper, Bruker Daltonik GmbH, Bremen, Germany) was used for species identification according to the manufacturer’s instructions. For DNA extraction, appropriate biomass was collected and re-suspended in 1.5 mL Eppendorf tubes in 1 mL 0.9% sodium chloride (NaCl). The tubes were centrifuged at 8,000 rpm for 1 min, and the supernatant was discarded. The remaining pellets were treated with a mixture of lysozyme and mutanolysin (LM) and incubated at 37°C for 30 min to disrupt cell walls. Then, genomic DNA was extracted using the QIAamp^®^ DNA Mini Kit (Qiagen, Hilden, Germany) according to the manufacturer’s instructions. The *sodA* gene was amplified and sequenced for 60 streptococcal isolates obtained from saliva and fecal samples of 13 IBD patients using the forward primer *sodA*-F 5′-TRCAYCATGAYAARCACCAT-3′ and the reverse primer *sodA*-R 5′-ARRTARTAMGCRTGYTCCCARACRTC-3′ as previously described (Conrads et al., 2017). PCR was performed under the following conditions: an initial denaturation step at 94°C for 2 min, followed by 35 cycles (each cycle consisting of 94°C for 30 s, 50°C for 30 s, and 72°C for 1 min), terminated by a final extension step at 72°C for 10 min. The *sodA* gene sequences were analyzed with the GenBank using BLAST (Altschul et al., 1990) to accurately identify the streptococcal species of the 60 investigated isolates. For *sodA* phylogenetic tree reconstruction, a multi-fasta file of *sodA* gene sequences was aligned using the ClustalW algorithm implemented in MEGA version 11 software (Tamura et al., 2021). Subsequently, the multiple sequence alignments were used to reconstruct a maximum likelihood (ML) phylogenetic tree using the default settings and the 500 replicates bootstrap test in MEGA11.

## Results

### Characteristics of the study groups

In total, we enrolled 14 IBD patients (UC, n = 7; CD, n = 7) and 12 healthy controls (**Table 1**). Only four patients had an active phase of UC, whereas the remaining 10 patients were in the remission phase (UC, n = 3; CD, n = 7). There were no significant differences in age (*p* = 0.7518) or gender (*p* = 0.4312) between IBD and control groups. All demographic data of the study groups and the clinical data of the IBD patients are shown in **Table 1**.

### Salivary microbiome alterations in IBD patients

**Figure 1:**
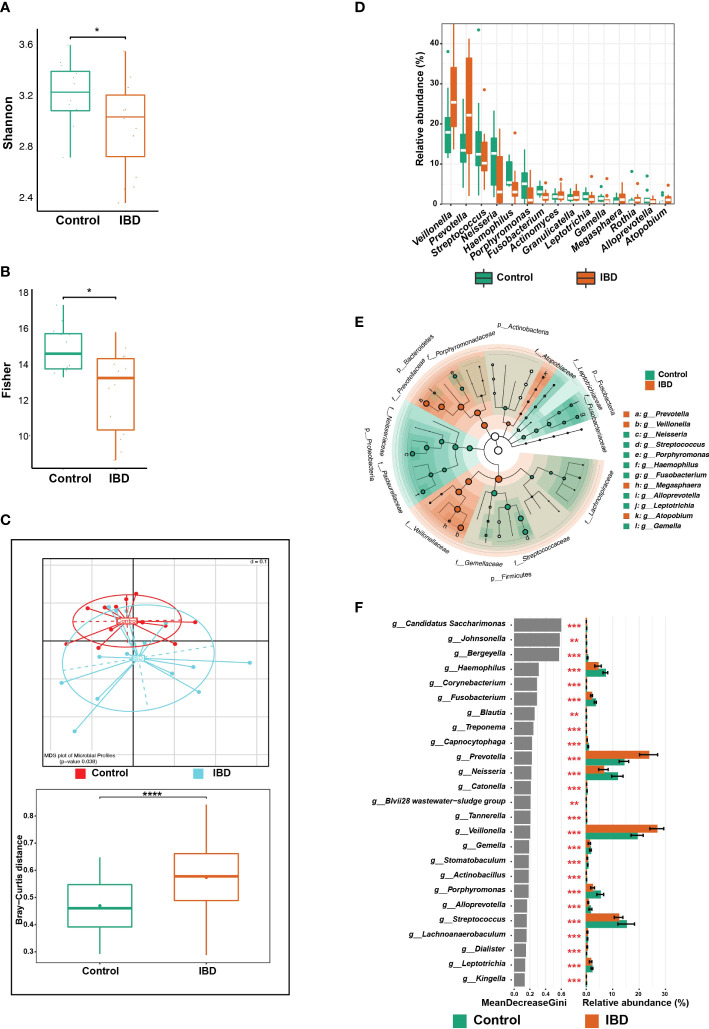
Changes in the salivary microbiome of IBD patients compared to healthy controls. **(A)** Boxplots show alpha diversity analysis performed on 15 saliva samples from 14 IBD patients (including one additional sample from P01) and 12 from 12 control subjects. **(B)** Boxplots showing the significance of Fisher’s test comparing the alpha diversity of the two groups without the additional sample from P01. **(C)** Beta diversity analysis represented by multidimensional scaling (MDS) and pairwise Bray-Curtis distances (boxplots). **(D)** Boxplots showing differentially abundant genera. **(E)** LEfSe cladogram demonstrating the phylogenetic relationship among genera that were enriched in both groups, where the dot size represents the mean abundance of the genus. **(F)** The top 25 significant genera were identified using random forest models, ranked by the index of accuracy and Gini. The symbols (*), (**), (***), and (****) indicate the significance values of *P* < 0.05, *P* < 0.01, *P* < 0.001, and *P* < 0.0001, respectively.

In the 27 sequenced saliva samples (15 IBD [including the additional sample from P01] and 12 controls), an average of 19,880 (median = 16,760) high-quality and chimera-tested sequences were analyzed, representing a total of 137 OTUs. Alpha diversity analysis showed that the number of detected taxonomic groups i.e., Shannon index, was significantly lower in samples from the IBD group (n = 15) compared with the control group (n = 12) (**Figure 1A**). Exclusion of the first saliva sample from P01 revealed that the richness and Shannon index of the IBD group were still lower compared with the control group, but the statistical significance vanished (**Figure S1A**), except for Fisher’s test (**Figure 1B**), suggesting that more saliva samples are needed to achieve significance for all statistical tests. To avoid any statistical bias, we included only one sample per patient (i.e., only the last sample).

**Figure 2:**
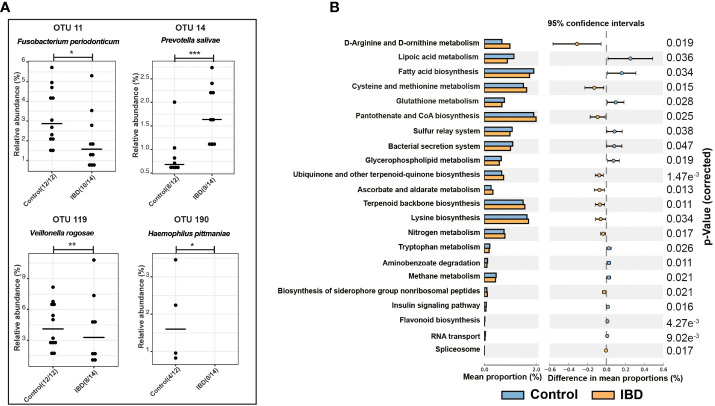
Differential OTUs abundances and metabolic functions of the salivary microbiome. **(A)** Significantly abundant OTUs that were increased in the salivary microbiome of IBD patients and healthy controls and their representative species according to BLAST analysis. **(B)** PICRUSt analysis showing KEGG pathways significantly enriched in the salivary microbiome of IBD and healthy controls. The symbols (*), (**), and (***) indicate the significance values of *P* < 0.05, *P* < 0.01, and *P* < 0.001, respectively.

The overall microbial composition of saliva was altered in the IBD patients, compared to the control subjects. After controlling for age, and gender, and including only one sample from P01, beta diversity analysis showed significant differences among the IBD and control groups (*p* = 0.038; **Figure 1C**). To further support this finding, we compared the distances between the two groups using the Bray–Curtis dissimilarity method, which confirmed that the IBD group had the highest beta diversity heterogeneity, while the control group had the lowest (*p* < 0.0001; **Figure 1C**). In addition, we analyzed the variation in salivary microbial taxa at all taxonomic levels among IBD and control groups, and we identified six phyla that showed marked changes across the two groups. The phyla Firmicutes, Bacteroidetes, and Proteobacteria dominated the salivary microbiome of both groups. We detected an increased abundance of Firmicutes, Bacteroidetes, and Actinobacteria, which was accompanied by depletion of Proteobacteria, Fusobacteria, and Patescibacteria in IBD patients compared to the control subjects (**Figure S1B**). At the genus level, the five genera *Veillonella*, *Prevotella*, *Megasphaera*, *Atopobium*, and *Rothia* were highly abundant (median: 25.4%, 22.2%, 1.2%, 1.1% and 1%, respectively) in IBD compared to healthy controls (17.9%, 13.4%, 0.6%, 0.6% and 0.4%, respectively) (**Figures 1D, E**). In particular, the two genera *Veillonella* and *Prevotella* were significantly more abundant in IBD, accounting for up to 47.6% of the total salivary microbiome (**Figures 1D, F**). On the other hand, at least 17 genera were significantly more abundant in controls compared with IBD patients and were therefore associated with a healthy gut state (**Figure 1F**). Among these 17 genera, only the five genera *Neisseria* (12.7%), *Streptococcus* (12.5%), *Haemophilus* (5.4%), *Porphyromonas* (5.1%), and *Fusobacterium* (3.1%) predominated and accounted for 38.8% of the salivary microbiome in controls, whereas in IBD patients they accounted for only 18.9% (3%, 10.2%, 3%, 1%, and 1.7%, respectively) (**Figures 1D, F**). Only four OTUs (OTU 11, 14, 119, and 190) were significantly different between the IBD patients and controls (**Figure 2A**). To assign these OTUs to bacterial species, we compared their sequences to the 16S rRNA sequences database using BLAST; all results are shown in **Table S1**. The results of BLAST analysis revealed that OTU 14, which was significantly more abundant in IBD patients, belonged to *Prevotella salivae* (**Figure 2A**). On the other hand, OTUs 11, 119, and 190, which were significantly increased in controls, belonged to *Fusobacterium periodonticum*, *Veillonella rogosae*, and *Haemophilus pittmaniae*, respectively (**Figure 2A**).

### Changes in metabolic functions of the salivary microbiome

Using PICRUSt and the KEGG database, we predicted the functional composition of the salivary microbiome, which revealed changes in the metabolic functions between IBD and control groups (**Figure 2B**). In addition, we detected 22 KEGG metabolic pathways that were significantly altered between both groups (**Figure 2B**). Ten pathways were highly enriched in IBD, whereas 12 pathways were associated with healthy controls. Changes in the salivary microbiota of IBD patients affected amino acid metabolism, followed by cofactor biosynthesis and energy metabolism (**Figure 2B**). In contrast, fatty acids biosynthesis/metabolism, the sulfur relay system, and the bacterial secretion system were identified as essential bacterial metabolic functions that were highly enriched in the control group.

### The fecal microbiota of IBD patients harbor pathogenic taxa

**Figure 3:**
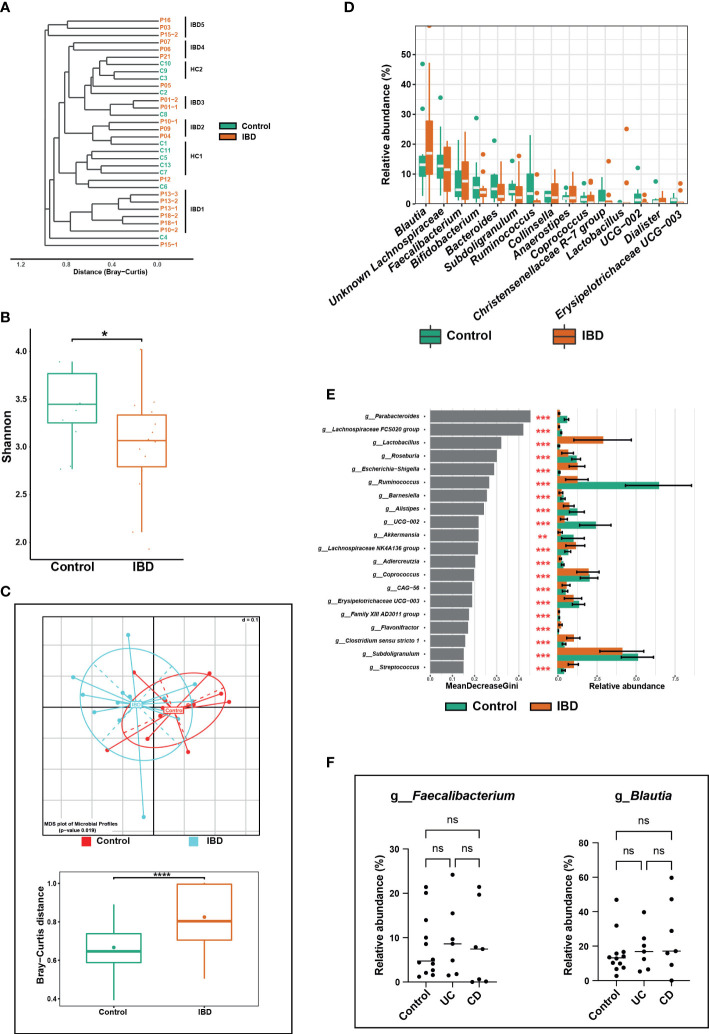
Alterations in the fecal microbiome of IBD patients compared to healthy controls. **(A)** Phylogenetic tree based on the microbiome distance between IBD patients and control subjects showing different clusters. **(B)** Shannon index representing alpha diversity of IBD (n = 14) and control subjects (n = 12). **(C)** Beta diversity analysis represented by multidimensional scaling (MDS) and pairwise Bray-Curtis distances (boxplots) between both groups. **(D)** Boxplots showing differentially abundant genera. **(E)** The top 20 significant genera were identified using random forest models, ranked by the index of accuracy and Gini. **(F)** Comparison of relative abundance of the genera *Faecalibacterium* and *Blautia* between control, ulcerative colitis (UC), and Crohn’s disease (CD) groups. The symbols (ns), (*), (**), and (***) indicate the significance values of *P* > 0.05 (not significant), *P* < 0.05, *P* < 0.01, and *P* < 0.001, respectively.

A total of 32 fecal samples (20 IBD [including six additional samples] and 12 controls) were analyzed. After reads quality filtering, an average of 15,617 (median = 16,582) high-quality sequences that were tested for chimeras were analyzed and revealed a total of 263 OTUs. Our cluster similarity analysis revealed two distinct clusters of controls (HC1 and HC2) and five for IBD (IBD1-5) samples, whereas no specific clustering related to IBD type or disease activity was detected in IBD patient samples (**Figure 3A**). As expected, the additional fecal samples from P01 (P01-2), P13 (P13-2 and P13-3), and P18 (P18-2) clustered with their initial samples (P01-1, P13-1, and P18-1, respectively), suggesting persistent microbial composition over time (**Figure 3A**). In contrast and interestingly, for patients P10 and P15, the second samples (P10-2 and P15-2) clustered far apart from their first sample (P10-1 and P15-1, respectively) (**Figure 3A**). Similar to the salivary microbiome, we included only one sample per patient (i.e., only the last sample) to avoid any statistical bias.

IBD patients had significantly lower fecal microbial richness and diversity compared with controls, which was confirmed by calculating the effective number of Simpson and Shannon indices (**Figure 3B**). The significant differences in fecal microbial community structure between IBD and control groups were confirmed by several beta diversity indices, which indicated greater heterogenicity in the microbial communities of IBD samples compared with their control counterparts (**Figure 3C**).

**Figure 4:**
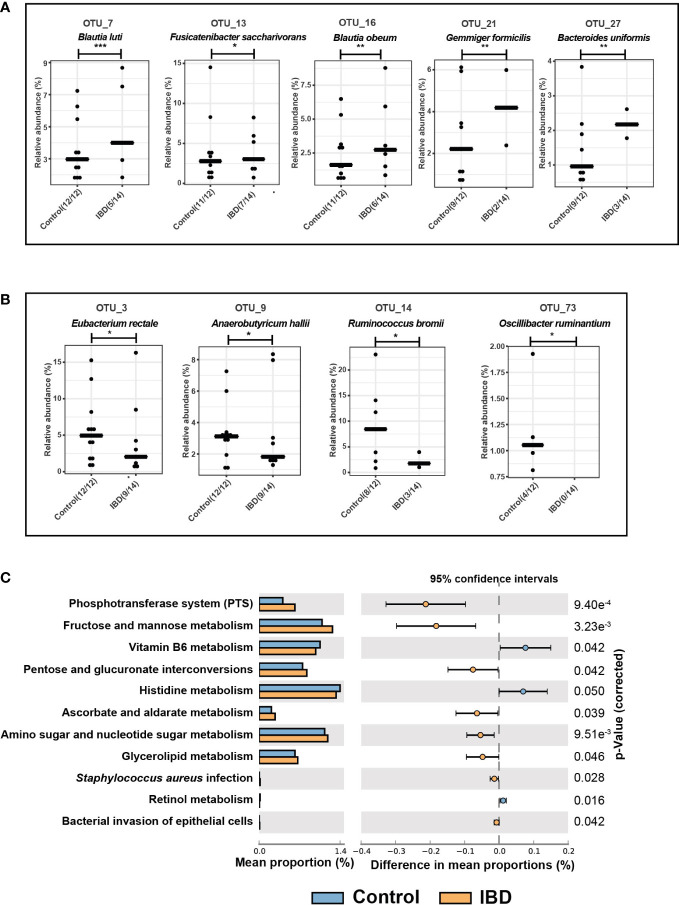
Differential OTUs abundances and metabolic functions of the fecal microbiome. **(A)** Significantly increased OTUs in the fecal microbiome of IBD patients and their representative species according to BLAST analysis. **(B)** Significantly increased OTUs in the fecal microbiome of healthy controls and their assigned species. **(C)** PICRUSt analysis showing KEGG pathways significantly enriched in the fecal microbiome of IBD and healthy controls. The symbols (*), (**), and (***) indicate the significance values of *P* < 0.05, *P* < 0.01, and *P* < 0.001, respectively.

The phyla Firmicutes, Actinobacteria, and Bacteroidetes were most represented in both groups, whereas Proteobacteria had less contribution but was significantly increased in IBD (2.1%) compared to the control group (0.8%) (**Figure S1C**). Although the IBD patients had a higher relative abundance of Firmicutes and Actinobacteria and a lower abundance of Bacteroidetes compared with the control group, these differences between the two groups were not significant. At the genus level, *Blautia*, unknown *Lachnospiraceae*, *Faecalibacterium*, *Bifidobacterium*, *Bacteroides*, *Subdoligranulum*, *Ruminococcus*, and *Collinsella* were the predominant genera and together they accounted for 51.4% and 46.7 of the fecal microbiomes of healthy control and IBD groups, respectively (**Figure 3D**). Genera that significantly altered among IBD and control groups are shown in **Figure 3E**. For example, the relative abundances of *Subdoligranulum* (2.1% vs. 4.1%), *UCG-002 - Oscillospiraceae* (0.8% vs. 2.9%), *Coprococcus* (0.4% vs. 1.5%), *Roseburia* (0.2% vs 0.9%), and *Ruminococcus* (0.0% vs. 3.5%) were significantly lower in the IBD group compared to the control group (**Figure 3E**). Whereas the relative abundances of the genera *Clostridium sensu stricto 1* (1.8% vs 0.9%), *Streptococcus* (0.5% vs. 0.2%), *Escherichia-Shigella* (0.5% vs. 0.0%), and *Lactobacillus* (0.03% vs 0.0%) were significantly higher in the IBD group compared with the control group (**Figure 3E**). In contrast to previous studies (Willing et al., 2010; Bourgonje et al., 2022), the abundances of the genera *Blautia* and *Faecalibacterium* were higher in the IBD group (17.0% and 7.6%, respectively) than in the control group (13.1% and 4.7%, respectively) (**Figure 3D**). However, this high abundance was not significant and was associated neither with UC nor CD conditions (**Figure 3F**). In total, five OTUs (OTU 7, 13, 16, 21, and 27) were significantly increased in the IBD group (**Figure 4A**), while only four OTUs (OTU 3, 9, 14, and 73) were significantly increased in the control group (**Figure 4B**). The results of BLAST analysis revealed that OTU 7, 13, 16, 21, and 27, which were significantly increased in IBD patients, belonged to *Blautia luti*, *Fusicatenibacter saccharivorans*, *Blautia obeum*, *Gemmiger formicilis*, and *Bacteroides uniformis*, respectively (**Figure 4A** and **Table S1**). In contrast, OTU 3, 9, 14, and 73 that were significantly elevated in the control group, were assigned to *Eubacterium rectale*, *Anaerobutyricum hallii*, *Ruminococcus bromii*, and *Oscillibacter ruminantium*, respectively (**Figure 4B** and **Table S1**).

### The metabolic functions of the fecal microbiome

The functional composition of the fecal microbiome of IBD patients and control groups was compared using KEGG pathway analyses. The pathways of Vitamin B6, histidine, and retinol metabolism were significantly downregulated in the gut microbiome of IBD patients (**Figure 4C**) compared to the controls. In addition, the following metabolic pathways were significantly enriched in the IBD group: phosphotransferase system (PTS), fructose and mannose metabolism, pentose and glucuronate interconversions, ascorbate and aldarate metabolism, amino sugar and glycerolipid metabolism (**Figure 4C**). Interestingly, human disease pathways associated with *Staphylococcus aureus* infection, and bacterial invasion of epithelial cells were significantly linked with IBD patients (**Figure 4C**).

### The Oral-gut axis: shared genera with an oral origin

**Figure 5:**
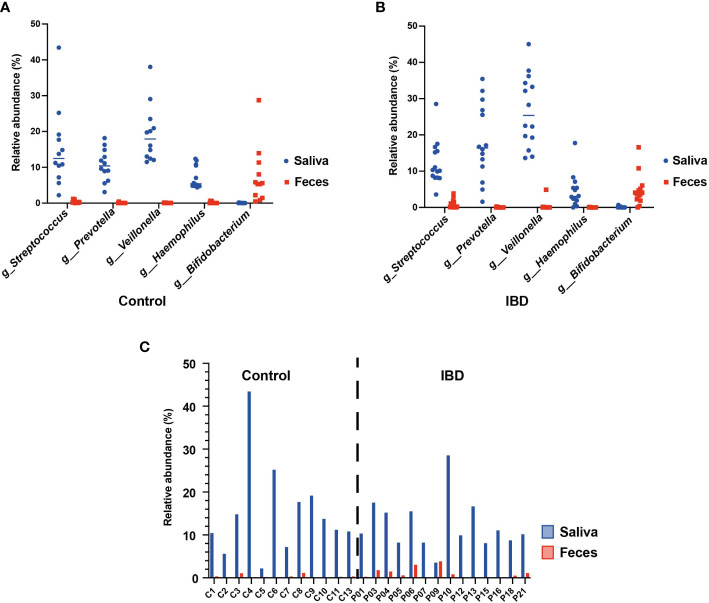
The oral-gut axis: core oral taxa (*Streptococcus*, *Prevotella*, *Veillonella*, *Haemophilus*, and *Bifidobacterium*) detected in the 16S rRNA sequences of the salivary and fecal microbiome. **(A)** comparison between the salivary and fecal microbiome of healthy controls. **(B)** comparison between the salivary and fecal microbiome of IBD patients. **(C)** Individual comparison between the relative abundance of *Streptococcus* from the salivary and fecal microbiome of healthy controls and IBD patients.

Several studies suggested frequent colonization of the gut by oral commensals belonging to specific genera such as *Streptococcus*, *Prevotella*, *Veillonella*, *Haemophilus*, and *Bifidobacterium* (Schmidt et al., 2019; Kitamoto et al., 2020; Hu et al., 2021). To test this hypothesis, we compared the relative abundances of these genera in the 16S rRNA sequence datasets of saliva and fecal samples from the IBD and control groups. Our analysis revealed that the relative abundances of these genera indeed differed greatly between the two habitats (**Figures 5A, B**). For example, *Veillonella*, *Prevotella*, and *Haemophilus* were detected only in the saliva of IBD patients (25.4%, 16.4%, and 3%, respectively) and the control group (17.4%, 10.4%, and 5.4%, respectively) but not in their feces. In contrast, *Bifidobacterium* was detected only in the feces of IBD (3.9%) and control subjects (5.4%). Interestingly, only the genus *Streptococcus* was detected in both habitats of IBD (saliva = 10.2% and feces = 0.5%) and control groups (saliva = 12.5% and feces = 0.2%). Furthermore, the abundance of streptococci in the feces of IBD patients was significantly increased and patient-dependent, with no fecal streptococci detected in patients P15 and P16 (**Figure 5C**) as the only exceptions. However, to accurately understand the microbial colonization of both habitats, it is necessary to track the presence of viable bacterial cells at the resolution of species and strain levels, rather than detecting only sequences at the DNA level that may not be supported by living cells. Therefore, we isolated and identified the oral and intestinal streptococci from saliva and fecal samples of IBD and control groups using culture-based methods. In total, 57 and 91 strains of streptococci were isolated from the saliva samples, as well as 40 and 31 strains were isolated from the fecal samples of the control and IBD groups, respectively (**Table S2**). MALDI-TOF MS identification assigned the salivary strains to 15 different streptococcal species, whereas 12 streptococcal species were detected in the fecal samples. Consistent with the sequence-based findings, we did not detect any streptococcal strain in the fecal samples from P15 and P16, including the additional samples. Furthermore, *S. parasanguinis* was the most common streptococcal species colonizing both habitats (saliva n = 30; feces n = 23) (**Table S2**). However, MALDI-TOF MS results regarding the species identification of *S. mitis*, *S. oralis*, *S. peroris*, *S. pneumoniae*, and *S. infantis* were frequently not distinct, giving different species as first- and second-best matches, often with score values below 2.0.

**Figure 6:**
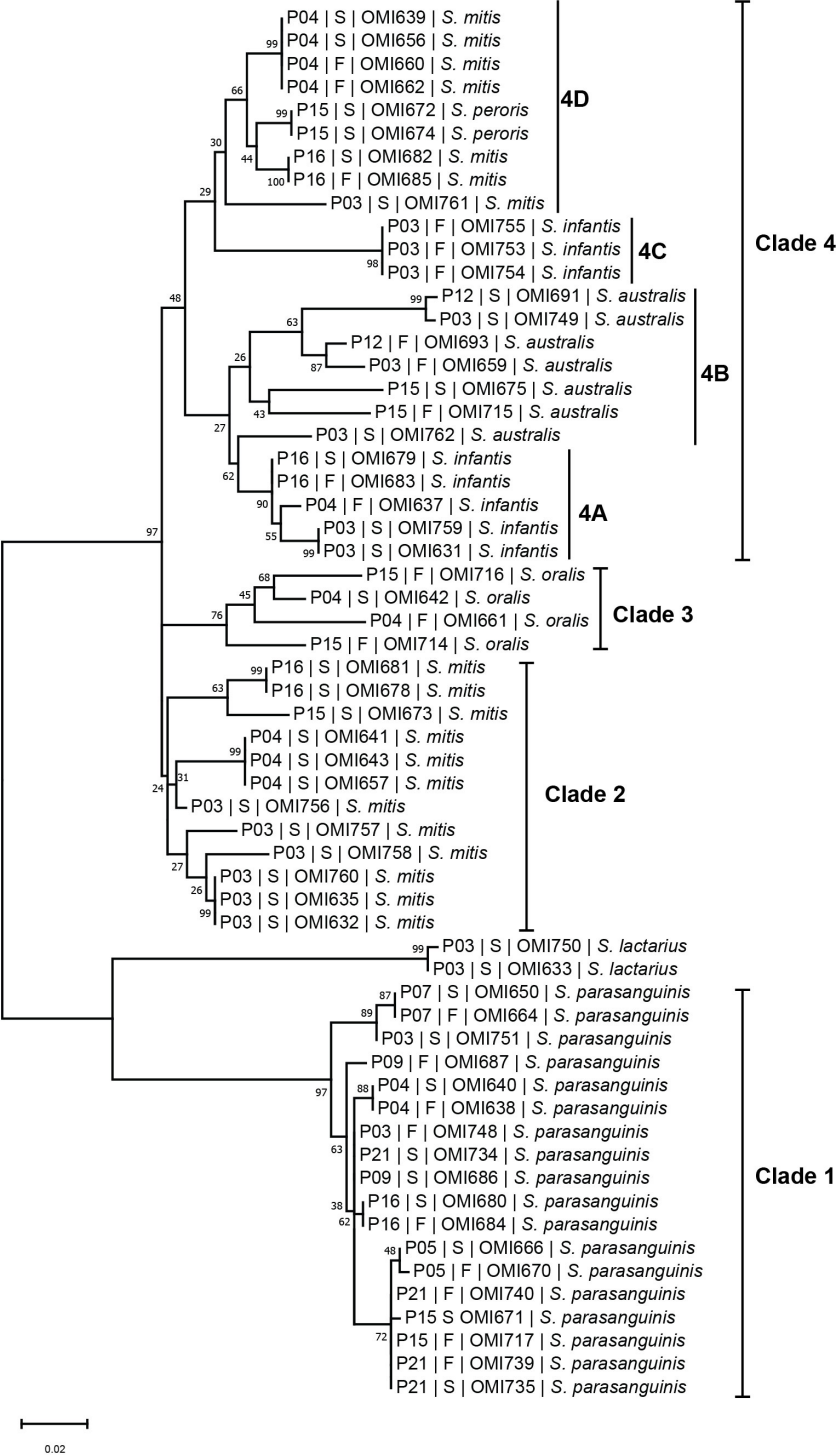
The streptococcal *sodA* sequence-based phylogenetic tree. Mid-rooted maximum likelihood phylogenetic tree based on *sodA* gene sequences of 60 streptococcal strains isolated from saliva (“S”) and fecal (“F”) samples of IBD patients (“P”).

To investigate the phylogenetic relationship between the oral and intestinal streptococcal strains, we amplified and sequenced the highly informative *sodA* gene of a subset (n = 60) of streptococcal strains obtained from saliva and fecal samples of 13 IBD patients. *SodA* sequence BLAST analysis accurately identified the 60 investigated streptococcal isolates compared with MALDI-TOF MS (**Table S2**). The phylogenetic tree based on *sodA* sequences revealed four main species-specific clades (1-4). Clades 1, 2, and 3 contained isolates *S. parasanguinis*, *S. mitis*, and *S. oralis*, respectively (**Figure 6**), whereas clade 4 represented the largest clade with four subclades (4A, 4B, 4C, and 4D) including different streptococcal species. For instance, subclades 4A and 4C contained isolates belonging to *S. infantis*, while subclades 4B and 4D included isolates of *S. australis* and *S. mitis*, respectively, except for two *S. peroris* isolates in subclade 4D (**Figure 6**). Interestingly, we detected several patient-specific clusters comprising salivary and fecal streptococcal isolates collected from the same patient and belonging to the same species, suggesting that the oral cavity is an endogenous reservoir for intestinal strains. For example, *S. parasanguinis* isolates from saliva (OMI640, OMI666, OMI650, OMI680, and OMI735) and feces (OMI638, OMI670, OMI664, OMI684, and OMI739) of patients P04, P05, P07, P16, and P21, respectively, formed multiple patient-specific clusters within clade 1 and were distinct from other *S. parasanguinis* isolates derived from other patients (**Figure 6**). Similarly, the *S. infantis* isolates OMI679 (saliva) and OMI683 (feces) collected from P16 clustered within 4A and were well separated from OMI637 collected from P04.

## Discussion

This study aimed to characterize and compare the salivary and fecal microbiota of IBD patients and control subjects as well as to explore the oral-gut axis. One of the main findings was the identification of a signature of the salivary microbiome associated with IBD patients, which was mainly linked to a high abundance of the genera *Prevotella* and *Veillonella* and a depletion of the salivary genera *Streptococcus*, *Haemophilus*, and *Neisseria*, which are associated with the healthy gut state. These findings are consistent with previous studies showing similar results in the saliva of IBD patients from different Asian geographic regions (China and Japan) (Said et al., 2014; Xun et al., 2018). For example, Said et al. showed that the abundance of *Prevotella* and *Veillonella* was significantly increased in both UC and CD patients, whereas the abundance of the genera *Streptococcus*, *Haemophilus*, and *Neisseria* was significantly reduced, the latter only in the CD group (Said et al., 2014). In addition, the authors demonstrated that the relative abundance of *Prevotella* and *Veillonella* was associated with inflammatory marker levels such as lysozyme and IL-1ß. Several studies showed that an increased abundance of *Prevotella* and *Veillonella* in the oral cavity was associated not only with oral diseases such as periodontitis (Yamashita and Takeshita, 2017) but also with various systematic diseases such as rheumatoid arthritis (Kroese et al., 2021), lung (Segal et al., 2013), and esophagus diseases (Park and Lee, 2020). Furthermore, recent studies have revealed that members of both genera were characteristically abundant in the oral microbiota of patients with long-standing COVID-19 symptoms (long COVID) (Haran et al., 2021), and of patients at clinical high risk (CHR) for psychosis before the first episode of schizophrenia (Qing et al., 2021). Our findings were also supported at the species level, especially for *Prevotella*, as BLAST analysis revealed that OTU14, which was significantly increased in the IBD patients, represented *Prevotella salivae* (**Figure 2A**). *P. salivae* was previously isolated from the saliva of patients with chronic periodontitis (Sakamoto et al., 2004), and another study that characterized the salivary microbiome of children (3–4 years old) showed that the abundance of *P. salivae* was significantly higher in the caries-affected group than in the caries-free group (*P*<0.05) (Jiang et al., 2016). In addition, *P. salivae* was enriched in specimens of patients with infected root canals and periapical abscesses (Hsiao et al., 2012) and fresh tissue biopsies of oral squamous cell carcinoma patients (Perera et al., 2018). To the best of our knowledge, this is the first study showing a significant increase of *P. salivae* in the salivary microbiome of IBD patients.

Some *Prevotella* and *Veillonella* strains appear to be inflammophilic pathobionts that thrive in an inflammatory environment and have a higher intrinsic capacity to stimulate Th17- and IL-6- mediated inflammation, respectively, than strictly commensal streptococcal strains (van den Bogert et al., 2014; Larsen, 2017). It has been previously shown that transfer of *Prevotella*-rich dysbiotic gut microbiota from Asc knockout or NLRP6 knockout mice to wild-type mice induced experimental colitis characterized by increased weight loss, tissue pathology, and death in recipient mice (Elinav et al., 2011). Moreover, a previous study revealed that *P. salivae*, *P. melaninogenica*, and *P. nanceiensis* induced similar surface expression levels of CD83, CD86, and CD40 activation-makers compared with members of Proteobacteria (*Haemophilus influenzae* and *Moraxella catarrhalis*), but reduced production of IL-12p70, IL-23 and IL-10 cytokines in monocyte-derived dendritic cells (Larsen et al., 2012). However, it was hypothesized that the difference may be due to different lipopolysaccharide (LPS) structures, as *Prevotella* produce penta-acylated LPS, whereas *H. influenzae* and *M. catarrhalis* produce hexa-acylated and hepta-acylated LPS, respectively (Larsen et al., 2012; Larsen et al., 2015).

Of note, *Prevotella* and *Veillonella* are the most abundant oral nitrate- and sulfate-reducing bacteria. During denitrification, nitrate is first converted to nitrite and then (either enzymatically or spontaneously after acidification) to nitrogen oxides, including nitric oxide (NO), while the end product of sulfate reduction is H_2_S (González-Soltero et al., 2020). NO is known to act as a physiological mediator of cell-to-cell signaling in the regulation of cardiovascular function (e.g., blood pressure), immunity, and metabolism. Another example of reactive nitrogen species is the nitrosation of cysteine residues in proteins (formation of S-nitrosothiols) with functional consequences. Nitrosation of amines, on the other hand, leads to the formation of N-nitrosamines, a class of chemical compounds with putative carcinogenic properties. Similarly, nitration of proteins (i.e., incorporation of a NO_2_ group) can impair their function (Carlström et al., 2020). The isoform of NO synthase (iNOS) expressed by inflammatory cells has been shown to be upregulated in the colon of IBD patients, and dysbiosis in NO metabolism has been associated with UC conditions (Roediger, 2008; Vermeiren et al., 2012). Furthermore, it was reported that the excess production and prolonged exposure of bacterial NO in combination with sulfide lead to active colitis (Roediger, 2008). Similarly, excessive H_2_S production by the local microbiota has been associated with the initiation of inflammation and the pathophysiology of several diseases such as schizophrenia and colitis (Al Nabhani et al., 2019; Qing et al., 2021; Buret et al., 2022). The latter was triggered by the destabilization of the mucus layer by reducing S-S bonds in the protein network, which is toxic to colonic epithelial cells (Al Nabhani et al., 2019). Interestingly, we discovered significantly upregulated signaling pathways involved in bacterial nitrate (nitrogen metabolism) and assimilatory sulfate (cysteine and methionine metabolism) reduction in the salivary microbiome of IBD patients compared with control groups (**Figure 2B**). These findings suggest that the shift in salivary microbiota toward nitrate- and sulfate-reducing bacteria, such as *Prevotella* and *Veillonella*, might indicate a direct contribution of the oral microbiota to the development of IBD. Taken together, our results indicate an association between the salivary microbiome and IBD suggesting that *Prevotella* and *Veillonella* may be promising biomarkers for disease detection. However, the role of *P. salivae* in IBD has so far not been shown and should be further explored.

In the last decade, alterations in the fecal microbiome of IBD patients have been studied in detail and have been proposed as biomarkers for disease diagnosis and treatment response. We detected a lower microbial richness and diversity, as well as the phylum Proteobacteria was significantly increased in the fecal samples of IBD patients compared with healthy controls, consistent with the results of previously published studies (Willing et al., 2010; Vich Vila et al., 2018; Galazzo et al., 2019). In addition, we demonstrated that fecal samples collected at different time points from the same patient clustered together, indicating the stability of fecal microbiota composition over time. This is consistent with a previous study demonstrating the consistency of gut microbiota composition in individuals from different population cohorts (Zhernakova et al., 2016). Although no significant differences were observed, we detected an increase in the relative abundances of the genera *Blautia* and *Faecalibacterium* in IBD compared to the control groups. A previous study revealed that *Blautia* was increased in UC patients compared with healthy controls, and these patients had a higher abundance of *Faecalibacterium* compared to CD patients (Nishino et al., 2018). However, our results revealed no significant difference in the abundance of *Faecalibacterium* among UC and CD (**Figure 3E**), which might be due to the small sample size of each group (n = 7). Interestingly, we demonstrated that IBD patients had two OTUs (7 and 16) that were significantly increased compared with the control group and belonged to the genus *Blautia* (*B. luti* and *B. obeum*, respectively) (**Figure 4A**). These results suggest that the increased abundance of the genus *Blautia* was species-dependent. This is concordant with a previous report that recommended considering the importance of species differences rather than generalizing the effects of *Blautia* at the genus level (Liu et al., 2021b). Further studies are required to determine whether *Blautia* plays a direct regulatory role in disease development.

The genera *Clostridium sensu stricto 1* and *Escherichia-Shigella* were also significantly more abundant in IBD patients compared with control subjects. Members of these genera are important pathobionts that may play a role in the development of IBD. *Escherichia-Shigella* has been shown to adhere to the mucosal epithelial cells of the colon and positively correlate with high expression of pro-inflammatory cytokines that disrupt the integrity of the intestinal barrier (Xu et al., 2018). Moreover, *Clostridium sensu stricto 1* includes an opportunistic pathogen associated with intestinal inflammation (Chen et al., 2014). Interestingly, our results showed significant enrichment of metabolic pathways, including bacterial invasion of epithelial cells, in the fecal samples of IBD patients, indicating damage to the intestinal epithelium and consistent with the observed increase of *Clostridium sensu stricto 1* and *Escherichia-Shigella*. Therefore, restoring the abundance of *Clostridium sensu stricto 1* and *Escherichia-Shigella* may help restore the gut microbial composition in IBD patients.

Our 16S rRNA sequence analysis of the core oral taxa (*Streptococcus*, *Prevotella*, *Veillonella*, *Haemophilus*, and *Bifidobacterium*) in the salivary and fecal microbiome revealed that *Streptococcus* was the only common genus in both habitats and was significantly increased in fecal samples from IBD patients. This finding suggests that ectopic gut colonization by oral bacteria is increased in patients with IBD. In contrast to previously published studies based only on sequencing data, here we performed a culture-based analysis to determine the presence of viable streptococcal strains in the gut (Schmidt et al., 2019). We were able to isolate 57 and 91 streptococcal strains from saliva, whereas 40 and 31 strains were isolated from fecal samples from the control and IBD groups, respectively (**Table S2**). Remarkably, we show that oral and intestinal streptococcal strains isolated from the same patient and belonging to the same species were genetically related, suggesting transmission from the mouth to the gut with subsequent colonization. These findings strongly support our results based on the 16S rRNA sequences analysis and the previous observations of Schmidt et al. suggesting frequent transfer of oral strain populations to the gut (Schmidt et al.).

Among the strengths of this study is the investigation of the salivary and fecal microbiome and the oral-gut axis in IBD patients through combining molecular and culture-based approaches. However, the main limitation of the present study is the small sample size, which affected the power of alpha diversity analysis for the salivary microbiome. In addition, our study is restricted to the salivary microbiome, and the oral hygiene and dental status of the patients were not considered, so despite their potential value. Hence, no conclusion can be drawn about the relationship between the biofilm microbiome and IBD. More advanced methods such as whole-genome sequencing need to be applied to explore the aspect of within-patient evolutionary pathways that enable oral streptococcal strains to adapt to the intestinal habitat. Of note, IBD is characterized by heterogeneity along multiple clinical axes with overlapping phenotypes rather than a specific disease state, which may contribute to the patient-specific shifts in microbial composition that were detected in our study. Therefore, shotgun metagenomic sequencing is required to determine the actual microbial gene content in the salivary and fecal microbiomes to be able to translate our findings into clinical practice.

In conclusion, our study demonstrates a correlation between dysbiosis of the salivary microbiota and IBD, independent of recognized gut dysbiosis, and we confirmed a key bacterial signature, including the high abundance of *Prevotella* and *Veillonella*, that occurs in this disease status. We hypothesize that the dysbiotic salivary microbiota leads to an alteration in microbial metabolites that could trigger inflammation. However, further studies are needed to determine the particular role of *Prevotella* (*P. salivae*) and *Veillonella* species associated with IBD and to understand their impact on IBD pathogenesis. In the fecal samples of IBD patients, we detected a significant increase of the pathogenic bacteria *Clostridium sensu stricto 1* and *Escherichia-Shigella*, which was combined with significant enrichment of bacterial invasion metabolic pathways, indicating damage to the intestinal epithelium.

Furthermore, we provide new insights into the oral-gut microbiome axis and reveal a potential molecular link between the oral and intestinal streptococci strains suggesting the oral cavity as an endogenous reservoir for gut microbial strains including pathobionts.

## Data availability statement

The microbiome raw sequence data presented in the study are deposited in the National Center for Biotechnology Information (NCBI) repository, accession number PRJNA855620.

## Ethics statement

This study was approved by the Ethics Committee of the RWTH Aachen University Hospital (No. EK 069/19 for IBD patients and No. EK206/09 for healthy control subjects) and conducted in accordance with the Declaration of Helsinki. All participants signed a written informed consent before sample donation.

## Author contributions

MA conceptualization, funding acquisition, bioinformatics analyses, data curation, interpretation, visualization, and writing the original draft. MH and AD recruited the patients and healthy controls and collected their samples as well as their clinical information. AB performing experiments and data analysis. DK and CT revised the manuscript. GC data interpretation, funding acquisition revision, and editing of the manuscript. All authors read and approved the manuscript.

## Funding

This study was funded by the START Program of the RWTH Aachen University Hospital (*STREPTORANTES* #109/19). This work was supported by the CRC 1382 (Project-ID 403224013) and the IZKF projects OC1-6 and OC1-9.

## Acknowledgments

The authors thank all patients and healthy controls who contributed with samples to our study. We are grateful to Mrs. Beate Melzer-Krick for her excellent technical assistance. We would like to thank Mrs. Elena Recker for her help. The authors thank the ZIEL - Institute for Food & Health (Freising, Germany) for the 16S rRNA gene amplicon sequencing. The authors would like to thank Prof. Dr. E. Dahl, Dr. J. Wipperfürth, and members of the RWTH Aachen Biobank team for their kind support.

## Conflict of interest

The authors declare that the research was conducted in the absence of any commercial or financial relationships that could be construed as a potential conflict of interest.

## Publisher’s note

All claims expressed in this article are solely those of the authors and do not necessarily represent those of their affiliated organizations, or those of the publisher, the editors and the reviewers. Any product that may be evaluated in this article, or claim that may be made by its manufacturer, is not guaranteed or endorsed by the publisher.

## Supplementary material

Supplementary Figure 1 Additional information on the salivary and fecal microbiome alterations. **(A)** Shannon index representing alpha diversity analysis performed on 14 salivary samples from IBD patients and 12 from control subjects. **(B)** Boxplots showing the relative abundance of phyla detected in the salivary microbiome of IBD (n = 14) and control (n = 12) groups. **(C)** Boxplots showing the relative abundance of phyla detected in the fecal microbiome of IBD (n = 14) and control (n = 12) groups.
