# Supplementary figures and images for: The oral-gut axis: Salivary and fecal microbiome dysbiosis in patients with inflammatory bowel disease
Source: Front Cell Infect Microbiol. 2022 Oct 7;12:1010853. doi: 10.3389/fcimb.2022.1010853 (PMC9585322; doi:10.3389/fcimb.2022.1010853)

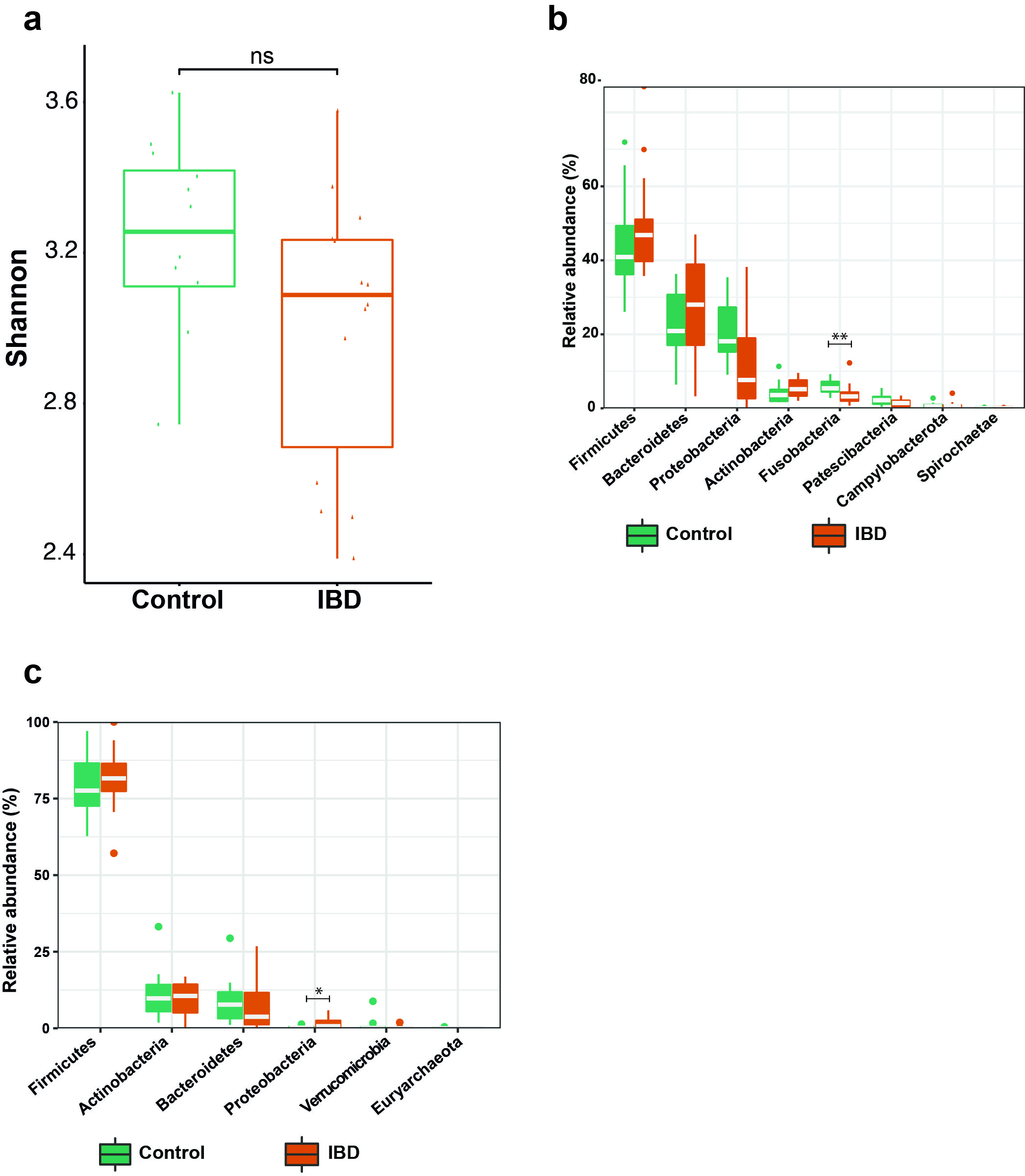

Supplement: Supplementary Figure 1 — Additional information on the salivary and fecal microbiome alterations. (A) Shannon index representing alpha diversity analysis performed on 14 salivary samples from IBD patients and 12 from control subjects. (B) Boxplots showing the relative abundance of phyla detected in the salivary microbiome of IBD (n = 14) and control (n = 12) groups. (C) Boxplots showing the relative abundance of phyla detected in the fecal microbiome of IBD (n = 14) and control (n = 12) groups. [file Image_1.jpeg]
